# Properties of Two Broad Host Range Phages of *Yersinia enterocolitica* Isolated from Wild Animals

**DOI:** 10.3390/ijms222111381

**Published:** 2021-10-21

**Authors:** Jens A. Hammerl, Andrea Barac, Philipp Erben, Julius Fuhrmann, Ashish Gadicherla, Franziska Kumsteller, Anne Lauckner, Felix Müller, Stefan Hertwig

**Affiliations:** Department of Biological Safety, German Federal Institute for Risk Assessment, Max-Dohrn Str. 8-10, D-10589 Berlin, Germany; Jens-Andre.Hammerl@bfr.bund.de (J.A.H.); Andrea.Barac@bfr.bund.de (A.B.); Philipp.Erben@live.de (P.E.); JuliusFuhrmann@hotmail.com (J.F.); Ashish.Gadicherla@bfr.bund.de (A.G.); Franziska.Kumsteller@gmail.de (F.K.); Lauckner-a@web.de (A.L.); Felix.Mueller1@gmx.de (F.M.)

**Keywords:** *Yersinia enterocolitica*, phage, genome, application, therapy

## Abstract

*Yersinia* (*Y*.) *enterocolitica* and *Y. pseudotuberculosis* are important zoonotic agents which can infect both humans and animals. To combat these pathogens, the application of strictly lytic phages may be a promising tool. Since only few *Yersinia* phages have been described yet, some of which demonstrated a high specificity for certain serotypes, we isolated two phages from game animals and characterized them in terms of their morphology, host specificity, lytic activity on two bio-/serotypes and genome composition. The T7-related podovirus vB_YenP_Rambo and the myovirus vB_YenM_P281, which is very similar to a previously described phage PY100, showed a broad host range. Together, they lysed all the 62 tested pathogenic *Y. enterocolitica* strains belonging to the most important bio-/serotypes in Europe. A cocktail containing these two phages strongly reduced cultures of a bio-/serotype B4/O:3 and a B2/O:9 strain, even at very low MOIs (multiplicity of infection) and different temperatures, though, lysis of bio-/serotype B2/O:9 by vB_YenM_P281 and also by the related phage PY100 only occurred at 37 °C. Both phages were additionally able to lyse various *Y. pseudotuberculosis* strains at 28 °C and 37 °C, but only when the growth medium was supplemented with calcium and magnesium cations.

## 1. Introduction

The genus *Yersinia* is currently composed of 28 species, three of which are known to be pathogenic for humans (https://lpsn.dsmz.de/genus/yersinia; access date: 22 May 2021). While *Yersinia* (*Y.*) *pestis* is the causative agent of plaque, *Y. enterocolitica* and *Y. pseudotuberculosis* are enteropathogenic species causing diseases termed yersiniosis [1]. In 2019, yersiniosis, which is mainly caused by *Y. enterocolitica,* was the fourth most commonly reported foodborne zoonotic disease in the European Union (EU) [2]. Typical symptoms are diarrhea (often bloody), fever and abdominal pain, which may be confused with appendicitis [3]. In Europe and many other countries, the presence of *Y. enterocolitica* is clearly associated with pigs [4,5]. Infections by this species may be caused by the consumption of raw or insufficiently cooked pork [6]. However, raw milk, water and vegetables like, e.g., mixed salad contaminated with *Y. enterocolitica* have also been reported as possible sources of infection [7,8,9]. Indeed, game animals that may contaminate food are known to contain this agent as well [10,11]. The species *Y. enterocolitica* is divided into six biotypes (1A, 1B, 2, 3, 4 and 5) and more than 70 serotypes [12]. In most European countries, strains belonging to bio-/serotype B4/O:3 prevail. Other common pathogenic bio-/serotypes are B2/O:9, B2/O:5,27 and, to a lesser extent, 1B/O:8. By contrast, biotype 1A strains, which frequently occur in the environment and food, are regarded as nonpathogenic. Those strains lack important virulence factors encoded by a chromosome or by a virulence plasmid (pYV) only existing in the other five biotypes, e.g., Ail (attachment-invasion locus), InvA (invasin) or *Yersinia* outer membrane proteins (Yops), some of which are toxins [13,14]. Even though *Yersinia* strains are not known to be resistant to a broad range of antibiotics (i.e., streptomycin, sulfonamide, trimethoprim/sulfamethoxazole, tetracycline, trimethoprim and chloramphenicol), methods are required to reduce these bacteria along the food chain. Most practical applications during slaughtering of pigs are intended to avoid contamination of carcasses, e.g., sealing off the rectum with a plastic bag, thermal inactivation of the bacteria and splitting of the carcass without the head [15,16,17]. One alternative approach to treat bacterial infections or to reduce pathogens in food is the application of virulent (strictly lytic) phages [18].

Phages are viruses exclusively infecting bacteria. They generally have a narrow host range and occur everywhere where their hosts live. Moreover, phages are also effective against multidrug-resistant bacteria and have a self-replicative mode of action. A number of phages infecting *Y. enterocolitica* has been described [19,20,21,22]. Most of them are podoviruses and myoviruses. To date, only two *Yersinia* siphoviruses have been reported, the well-characterized temperate phage PY54 and the virulent phage phiR2-01, which is similar to T5 but whose properties have not been thoroughly described yet [23,24,25,26,27]. Most podoviruses infecting *Y. enterocolitica* are related to T7 and have a rather narrow range as they mainly lyse O:3 strains [28,29,30]. On the other hand, some myoviruses exhibit a broader host range since they are additionally able to lyse strains of serotypes O:9 and O:5,27 [31,32], though some of these phages are active only at and below 25 °C [33]. One myovirus (fHe-Yen9-01) lysed 53 out of 81 (65.4%) *Y. enterocolitica* strains and was used for experiments with raw pork, ready-to-eat pork, milk and kitchen utensils leading to reductions of bacterial counts by 1 to 3 logs [31]. The widest host range of all *Yersinia* phages described thus far possesses phage PY100 isolated from farm manure in Germany [34]. PY100 not only infects various bio-/serotypes of *Y. enterocolitica*, but also *Y. pseudotuberculosis*, *Y. pestis*, as well as some other nonpathogenic *Yersinia* species. Interestingly, the very similar phage vB_Yen_X1, which features 99.5% sequence identity and 99% genomic coverage with PY100, was not able to lyse *Y. enterocolitica* O:9 strains [35].

In this work, we isolated and characterized two virulent phages from game animals, a podovirus (vB_YenP_Rambo) and a myovirus (vB_YenM_P281). We show that the phages revealed a very broad host range within the species *Y. enterocolitica* and may be well-suited for applications.

## 2. Results

### 2.1. The Phages Isolated from Game Exhibit Podovirus and Myovirus Morphology

**Figure 1:**
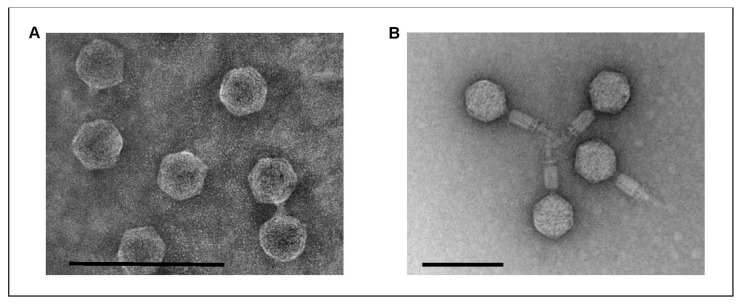
Electron micrographs of the phages vB_YenP_Rambo (**A**) and vB_YenM_P281 (**B**). The bar represents the size of 100 nm.

Phage vB_YenM_P281 was isolated from a female deer, whereas vB_YenP_Rambo was isolated from a male wild boar. Transmission electron microscopy (TEM) demonstrated that the phages are significantly different in terms of their morphology. Both possess an isometric head and a tail and thus belong to the order *Caudovirales* (Figure 1). However, while vB_YenP_Rambo has a very short tail and is clearly a podovirus, the tail of vB_YenM_P281 is long and contractile. Therefore, this phage is a myovirus.

### 2.2. vB_YenP_Rambo and vB_YenM_P281 Are Related to Known Phages

**Figure 2:**
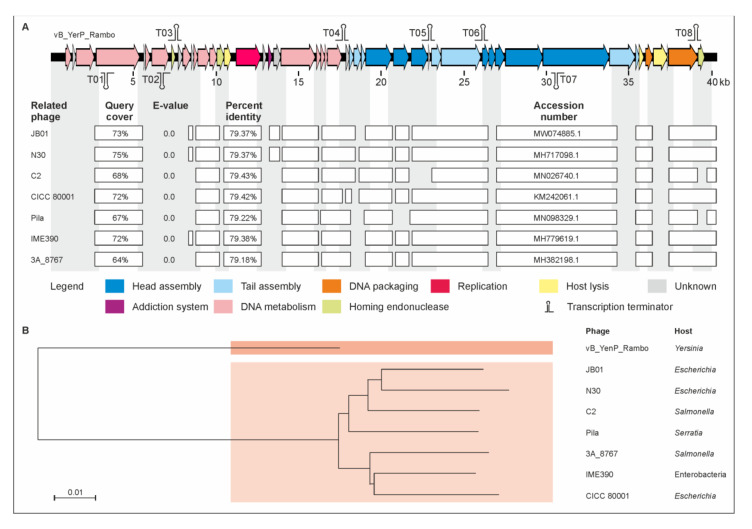
Gene map of phage vB_YenP_Rambo and its relationship to other podoviruses. (**A**) Gene map of the phage and identity values with other phages. White bars represent regions of high nucleotide similarity (>75%). (**B**) Phylogenetic tree (single nucleotide polymorphism (SNP)-based) of the phages. The scale bar represents the number of nucleotide substitutions per site.

To determine the relationship with other phages, the whole genome sequences of the phages were analyzed. The first one, vB_YenP_Rambo, has a linear genome of 40,327 bp with terminal direct repeats (199 bp), determined by run-off sequencing using primers binding approximately 200 bp away from the predicted ends of the genome, which is related to T7-like phages (Supplementary Material Table S1). The strongest similarities exist with the *Escherichia* phages JB01 and N30, to which vB_YenP_Rambo is approximately 79% identical over 73% and 75%, respectively, of their genomes (Figure 2). A slightly lower value was obtained with, e.g., the *Y. pestis* phage phiA1122 [36]. The majority of vB_YenP_Rambo gene products (gp), e.g., the RNA polymerase, the major capsid protein or the terminase (large subunit) are more than 80% identical to their counterparts in T7-like phages. One of the few gps that are significantly less similar (50% identical) is the tail fiber protein (TFP). While the first approximately 180 amino acids of the TFPs (gp17 in T7) of vB_YenP_Rambo, JB01 and phiA1122 are very similar, the downstream sequences including the region harboring the receptor-binding domain [37] share almost no sequence homology (Supplementary material Figure S1). This may explain the different host specificity of the three phages [37], though the vB_YenP_Rambo protein also shows the same dissimilarity when compared with the TFP of phiYeO3-12, a phage which specifically infects *Y. enterocolitica* O:3 strains [38].

**Figure 3:**
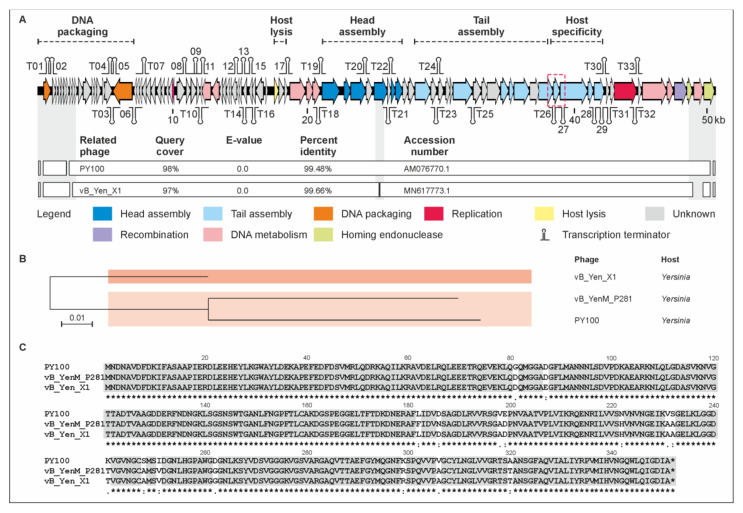
Gene map of phage vB_YenM_P281 and its relationship with PY100 and vB_Yen_X1. (**A**) Gene map of vB_YenM_P281 and values of identity with the other two phages. White bars represent regions of high nucleotide similarity (>75%). (**B**) Phylogenetic tree of the phages. The scale bar represents the number of nucleotide substitutions per site. (**C**) Alignment of tail fiber proteins 1 (ORF 78 (PY100), 78 (P281) and 69 (vB_Yen_X1)). The location of the vB_YenM_P281 tail fiber gene is indicated in the gene map (red box).

Phage vB_YenM_P281 has a genome of 50,481 bp, which is very closely related to that of the *Yersinia* phages PY100 (50,291 bp) and vB_Yen_X1 (48,848 bp), with which it is more than 99% identical over 98% and 97% of the genomes, respectively (Figure 3). Indeed, almost all the vB_YenM_P281 predicted gp’s were also identified in the other two phages. Since the small and large terminases of the phages are nearly identical, the start of the vB_YenM_P281 genome was chosen in accordance to that of PY100, which has been reported to pack its DNA by headful packing using a *pac* site as the initial start point [34]. Only at the end of the linear genomes there are a few open reading frames (ORFs) encoding hypothetical proteins or homing endonucleases which are less than 80% identical (Figure 3, Supplementary Material Table S2). Two vB_YenM_P281 ORFs (ORF07 and ORF90) encoding a hypothetical protein and a homing endonuclease, respectively, are even missing in vB_Yen_X1. For vB_Yen_X1, it has been reported that this phage lacks six PY100 ORFs (07, 47, 51, 58, 87, 91), whereas it would contain one ORF (ORF28) that does not exist in PY100 [35]. However, a closer look at the sequences revealed that this is not the case since apart from the PY100 ORFs 07 and 91 which correspond to the abovementioned ORFs 07 and 90 of vB_YenM_P281, all other the ORFs are present in the respective phage. The fact that also the tail fiber proteins of these phages are almost identical suggests that they have similar host specificities. Figure 3C shows alignment of tail fiber protein 1. Similar identity values of 98.9% (vB_Yen_X1) and 99% (PY100) over 100% and 92%, respectively, of the protein sequences were determined for tail fiber protein 2.

### 2.3. Phage vB_YenP_Rambo and vB_YenM_P281 Exhibited a Broad Host Range

**Figure 4:**
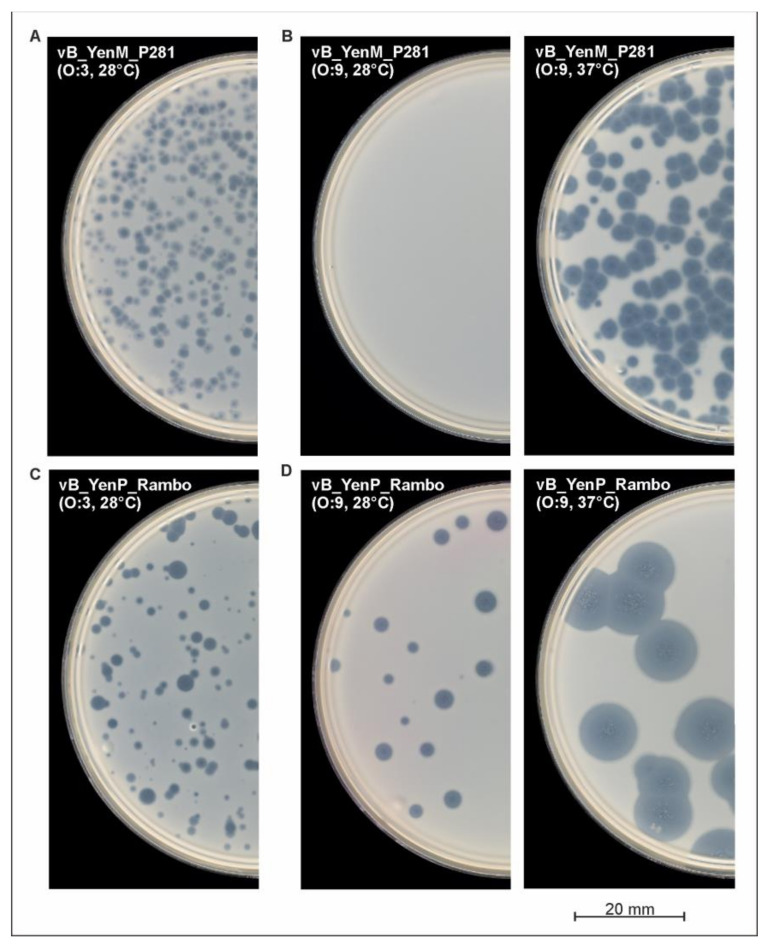
Plaque formation by the phages on lawns of *Y. enterocolitica* strongly depends on the temperature.(**A**) vB_YenM_P281 (O:3), (**B**) vB_YenM_P281 (O:9) (**C**) vB_YenP_Rambo (O:3), (**D**) vB_YenP_Rambo (O:9).

To determine host specificity of the three phages, 62 pathogenic *Y. enterocolitica* strains belonging to the bio-/serotypes B4/O:3, B2/O:9, B2/O:5,27 and 1B/O:8, which are mostly associated with yersiniosis [4], as well as 10 nonpathogenic biotype 1A strains were examined. The study revealed that the phages vB_YenP_Rambo and vB_YenM_P281 lysed almost all the B4/O:3 (Figure 4A,C) and B2/O:5,27 strains at 28 °C (Table 1). By contrast, no B2/O:9 strain was lysed by vB_YenM_P281 (Figure 4B). On the other hand, vB_YenP_Rambo did not show lytic activity on 1B/O:8. It is worth noting that similarly to vB_YenM_P281, phage PY100 also did not lyse any B2/O:9 strain at 28 °C. To elucidate whether the temperature may be important for the infection, the experiment was repeated at 37 °C. Table 1 shows that B4/O:3 and B2/O:5,27 strains were lysed by the phages at both temperatures but that the size of plaques increased significantly at 37 °C (in case of vB_YenP_Rambo, up to 8 mm in diameter, Figure 4D).

Much more importantly, at this temperature, both vB_YenM_P281 and PY100 were able to lyse 18 out of the 19 B2/O:9 strains (Figure 4B), while the same negative result as before was achieved with vB_YenP_Rambo and 1B/O:8. In conclusion, it can be stated that vB_YenP_Rambo lysed 80.6% of the pathogenic strains at 28 °C and 87% at 37 °C. On the other hand, 64.4% and 95% of these strains were susceptible to vB_YenM_P281, at 28 °C and 37 °C, respectively. Both phages together were able to lyse 95% of the pathogenic strains at 28 °C and 100% at 37 °C. Similar to the bio-/serotype B2/O:9, a strong lytic activity of the phages was observed with biotype 1A strains at 37 °C. While at this temperature, six and all the ten strains were lysed by vB_YenP_Rambo and vB_YenM_P281, respectively; a significantly lower lytic activity was detected at 28 °C (Table 1).

### 2.4. Lysis of Y. pseudotuberculosis by vB_YenM_P281 and PY100 Depends on the Presence of Calcium and Magnesium Cations

Phage vB_YenM_P281 is closely related to PY100, which has been reported to have a broad host range within the genus *Yersinia* [34]. This fact inspired us to also test and compare the lytic activity of vB_YenM_P281 and PY100 on the *Y. pseudotuberculosis* strains belonging to various serotypes (Table 2), though we did not observe any plaques of the phages on the 104 tested strains at both temperatures, 28 °C and 37 °C, in the NZCYM soft agar routinely used in our laboratory for this kind of experiment. In the study mentioned above, LB soft agar supplemented with 10 mM CaCl_2_ and 10 mM MgSO_4_ was used for plaque assays, which exclusively were carried out at 37 °C. We therefore supplemented NZCYM soft agar with CaCl_2_ and MgSO_4_ (20 mM each) and examined the lytic activity of the phages at both temperatures on the 55 selected strains representing the most important serotypes (Table 2). Surprisingly, plaques were obtained with vB_YenM_P281 and PY100 irrespective of the temperature. However, at 37 °C, more *Y. pseudotuberculosis* strains were lysed than at 28 °C (Table 2). On the contrary, phage vB_YenP_Rambo was not able to lyse *Y. pseudotuberculosis*, even under these conditions.

We finally studied the lytic activity of vB_YenM_P281 and vB_YenP_Rambo on reference strains of the 14 other *Yersinia* species (Table 3). Lysis by at least one phage was observed with *Y. bercovieri*, *Y. frederiksenii*, *Y. kristensenii* and *Y. wautersii*, but only at 37 °C. In some cases, the presence of CaCl_2_ and MgSO_4_ was necessary.

### 2.5. The Phages vB_YenP_Rambo and vB_YenM_P281 Show a Strong Lytic Activity

**Figure 5:**
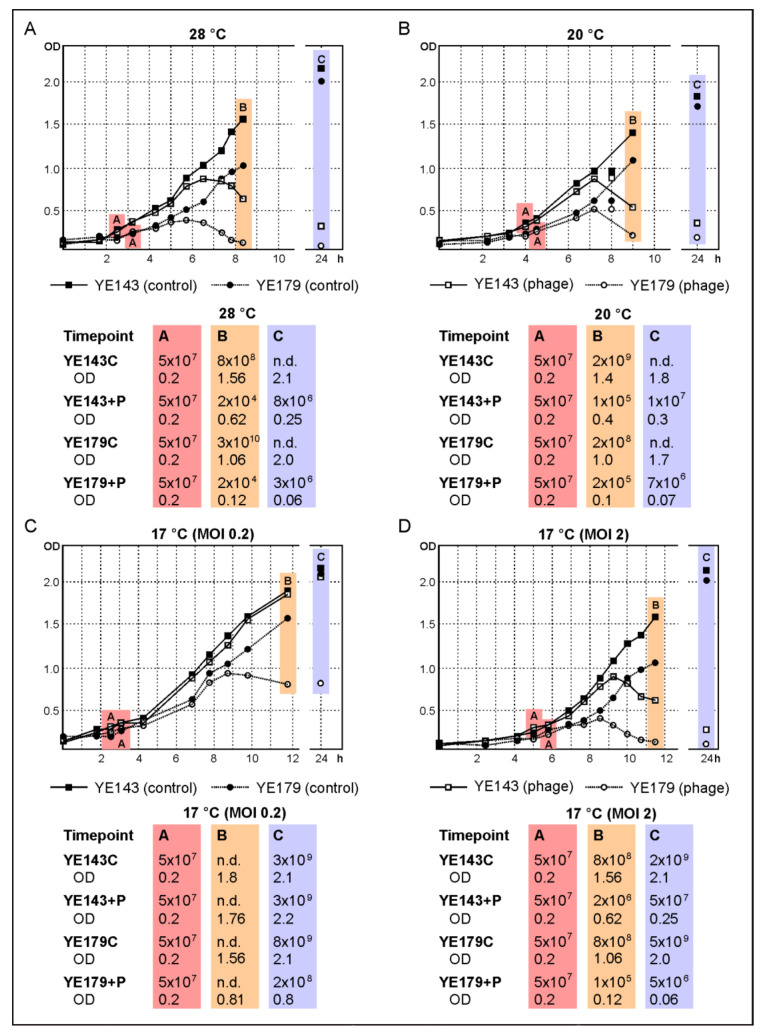
Reduction of the B4/O:3 strain YE179 and the B2/O:9 strain Y143/17 by a cocktail containing vB_YenP_Rambo and vB_YenM_P281 at different temperatures. Numbers of colony-forming units (CFU) determined at several timepoints: (**A**) CFU on addition of phage, 28 °C; (**B**) CFU at the end of day 1, 20 °C; (**C**) CFU after incubation overnight, 17 °C (MOI 0.2); (**D**) 17 °C (MOI 2). **n.d., not determined**.

Based on their wide host range, these two phages might be suitable to reduce *Y. enterocolitica* along the food chain, e.g., during the production of pork. Therefore, some lytic properties of a cocktail containing both phages were determined at various temperatures to determine the threshold at which the phages can be successfully applied since production may take place at different temperatures. We first studied the reduction of a bio-/serotype B4/O:3 (YE179) and a B2/O:9 (Y143/17) strain at 28 °C using an MOI of 0.2. Figure 5A shows that the phages significantly lysed both strains after 4–5 h. Indeed, determination of the cell numbers after approximately six hours of incubation with phages revealed a reduction of more than six (YE179) and more than four (Y143/17) orders of magnitude compared to the controls without the phage. Moreover, the optical density of the infected Y143/17 culture only slightly increased after incubation overnight, whereas the infected YE179 culture even remained at its level, suggesting a low rate of phage resistance. To examine the efficacy of lower MOIs, infections were repeated with diluted phage lysates. This study revealed MOIs down to 0.000001 to be sufficient for the lysis of both strains (OD of YE179 = 0.6 (control, 1.3), OD of Y143/17 = 0.2 (control, 1.4) after approximately four hours of incubation), which means a relation of one phage per million bacteria. In the next experiment, the lytic activity of the phages at lower temperatures was examined, applying the same conditions as at 28 °C. At 20 °C, a reduction of more than four (Y143/17) and three (YE179) orders of magnitude after 4–5 h of incubation was obtained (Figure 5B). To determine whether the surviving bacteria had become resistant against the phages within 24 h of incubation, the susceptibility of ten colonies isolated from the infected cultures of YE179 and Y143/17 was tested with each phage. All the twenty isolates were lysed by both phages indicating that the temperature (20 °C) did not induce resistance. The threshold temperature for efficient cell lysis within hours was 17 °C. However, at this temperature, a significantly better lysis was obtained after adding a tenfold number of phages (MOI 2). Using this MOI, a reduction of both strains of almost four (YE179) and, respectively, three (Y143/17) orders of magnitude was achieved (Figure 5D) within 5–6 h, whereas at a MOI of 0.2, only strain YE179 was reduced by almost two orders of magnitude after incubation overnight (Figure 5C).

## 3. Discussion

In this study, we isolated and characterized two *Y. enterocolitica* phages, vB_YenP_Rambo and vB_YenM_P281, which may be promising candidates for the biocontrol of this species. While vB_YenP_Rambo is a member of a large group of T7-like phages, to date, only two relatives of vB_YenM_P281 have been described, PY100 isolated from manure in Germany and vB_Yen_X1 recovered from sewage in China [34,35]. Remarkably, we isolated 13 very similar phages from game animals indicating that PY100-related phages are more common in nature than thought (Supplementary Material Figure S2). Together, vB_YenP_Rambo and vB_YenM_P281 lysed 95% (at 28 °C) and 100% (at 37 °C) of the 62 tested *Y. enterocolitica* strains belonging to the most important bio-/serotypes B4/O:3, B2/O:9, B2/O:5,27 and 1B/O:8. Moreover, the phages revealed a strong lytic activity and lysed strains under in vitro conditions even at rather low temperatures and at very low MOIs. Due to its extended host range, vB_YenP_Rambo differs from most other *Y. enterocolitica* podoviruses, which display a marked specificity for serotype O:3 [27]. Indeed, podoviruses are generally considered to have a narrower host range than myoviruses [39]. Thus, vB_YenP_Rambo is an exemption from this rule. Since myovirus vB_YenM_P281 is very closely related to PY100, we expected a similar host specificity. Surprisingly, however, our first studies on the host range of this phage disclosed significant differences from PY100. This mainly pertained to the lytic activity on *Y. enterocolitica* O:9 strains and *Y. pseudotuberculosis*, which were reported to be susceptible to PY100 [34], though our experiments showed that both vB_YenM_P281 and PY100 were only able to lyse *Y. enterocolitica* O:9 at 37 °C, but not at 28 °C, which has been suggested to be the optimal temperature for *Yersinia* [40]. Similarly, the closely related phage vB_Yen_X1 lysed all the relevant pathogenic serotypes of *Y. enterocolitica*, except for serotype O:9 [35]. We do not know at which temperature the cited study was performed, possibly below 37 °C. The host receptor for this group of phages has not been identified yet. Therefore, it can only be speculated whether the receptor of O:9 strains is exclusively available at 37 °C or whether the propagation of the phage is inhibited at lower temperatures. Regarding *Y. pseudotuberculosis,* the situation was different because not the temperature, but the presence of calcium and magnesium cations played the major role for cell lysis by vB_YenM_P281 and PY100 there, even though the highest lytic activity was obtained at 37 °C. Since NZCYM broth already contains MgSO_4_ (0.98 g/L), we assume that particularly the addition of calcium is important for infection. These ions are known to stimulate the adsorption of some phages [41,42,43]. Hence, adequate temperature and a sufficient amount of the cations should be considered when phages of the PY100 group are intended for the biocontrol of *Y. enterocolitica* O:9 and *Y. pseudotuberculosis*, respectively. Nevertheless, the combination of vB_YenM_P281 and vB_YenP_Rambo in a cocktail for the reduction of *Y. enterocolitica* is ideal because they complement each other excellently. While vB_YenP_Rambo lysed all the B2/O:9 strains, the same applied to vB_YenM_P281 and the bio-/serotype 1B/O:8. Our in vitro experiments also revealed that vB_YenP_Rambo is able to significantly reduce the cell number of a B2/O:9 culture at the temperatures at which vB_YenM_P281 is not active, even though a MOI of 2 was necessary at 17 °C. Comparison with the B4/O:3 strain suggests that the two phages act in concert so that a higher number of vB_YenP_Rambo is required to compensate for the inactivity of vB_YenM_P281 on B2/O:9 at this temperature. It would, of course, be advisable to supplement a cocktail by a phage like He-Yen9-, a T4-related myovirus that also exhibited a rather broad host range lysing 53/81 (65.4%) *Y. enterocolitica* strains, even though it was not active on bio-/serotype 1B/O:8 [31].

## 4. Materials and Methods

### 4.1. Bacterial Strains and Culture Conditions

All the bacterial strains of this study originate from the culture collection of the Consiliary Laboratory for *Yersinia* (KL *Yersinia*) hosted at the German Federal Institute for Risk Assessment (BfR), Berlin, Germany. If not otherwise indicated, *Yersinia* spp. bacteria were cultivated in/on lysogeny broth (LB)-based media at 28 °C. Cultivation in a liquid medium was conducted under continuous shaking at 200–225 rpm [44].

### 4.2. Isolation, Propagation and Purification of Phages

*Yersinia* phages described here originated from fecal samples from a deer and a wild boar hunted in northeast Germany. At KL *Yersinia*, 5 mL of SM buffer [45] was added to suspend the samples overnight at 4 °C. Afterwards, the material was subjected to centrifugation for 20 min at 8000 rpm and 10 °C. The supernatants were passed through 0.45 µm pore size filters (VWR International, Darmstadt, Germany) and stored until further processing at 4 °C. Determination of lytic activity was performed by spotting 10 µL of serial dilutions of each sample onto a lawn of *Y. enterocolitica* indicator strains belonging to various serotypes. After incubation overnight at 28 °C and room temperature (RT), the plaques were visually inspected and counted. Individual phages were purified by threefold recovery of single plaques. High-titer lysates of the phages were obtained by infecting 200 mL cultures of the indicator strain (OD_588_ = 0.5) with phages at a MOI of 0.01–0.1 or by preparing 10–20 agar plates with confluent lysis of the host bacteria. In this case, soft agar was harvested by scraping. Sixteen to eighteen hours after phage application, the lysates were centrifuged for 20 min at 10,000× *g* to remove the agar and debris and then filtered (see above). Phages were concentrated by ultracentrifugation and purified using CsCl step gradients as previously described [46].

### 4.3. Host Range Determination

The host range of purified phages was determined by spot activity assays. The respective indicator strain in the amount of 100 μL was mixed with 6 mL prewarmed NZCYM (VWR International, Darmstadt, Germany) soft agar (0.6%) and poured onto an LB agar plate [45]. Ten microliters of serial dilutions of each lysate (adjusted to ~1 × 10^7^ pfu/mL) were spotted onto the overlay agar and visually inspected after an overnight incubation at RT, 28 °C or 37 °C.

### 4.4. Transmission Electron Microscopy (TEM)

The CsCl-purified phages were investigated by means of TEM using the negative staining procedure with uranyl acetate (VWR International, Darmstadt, Germany) as previously described [46]. The specimens were examined by means of TEM using JEM-1010 (JEOL, Tokyo, Japan) at 80 kV acceleration voltage.

### 4.5. Phage DNA Preparation, Sequencing and Genome Annotation

For short-read, paired-end whole genome sequencing, phage DNA was extracted from concentrated virions by proteinase K/SDS treatment at 56 °C for 2 h followed by phenol chloroform extractions [45]. DNA sequencing libraries were prepared with a Nextera Flex DNA Sample Preparation Kit (Illumina, San Diego, CA, USA) according to the manufacturer’s protocol. Short-read paired-end sequencing was performed in 2 × 251 cycles on the Illumina MiSeq benchtop using a MiSeq Reagent v3 600-cycle Kit (Illumina). Raw reads were trimmed and de novo assembled using the in-house developed Aquamis pipeline, in which fastp and shovill (SPAdes-based) are included for trimming and assembly, respectively. Furthermore, it also includes mash (version 2.1) and quast (version 5.0.2) for reference search and quality control of the assemblies. Illumina sequencing resulted in single contigs consisting of 47,815 (10.9 Mb, sequencing depth > 250) and 54,736 reads (12.8 Mb, sequencing depth > 200) of vB_YenP_Rambo and vB_YenM_P281, respectively. For the prediction of putative coding sequences (CDS), the annotation tool of the PATRIC database was used. Further bioinformatics analysis (i.e., sequence comparison) was conducted using the blast suite (blastn, blastx, blastp; https://blast.ncbi.nlm.nih.gov/Blast.cgi; access date: 6 October 2021) of the National Center for Biotechnology Information (NCBI). Prediction of the potential transcription terminators was conducted using ARNold (http://rssf.i2bc.paris-saclay.fr/toolbox/arnold/; access date: 6 October 2021) [47,48]. The phage genomes were analyzed with ARAGORN (http://www.ansikte.se/ARAGORN/; accessed on 6 October 2021) and tRNAscan-SE 1.21 (http://lowelab.ucsc.edu/tRNAscan-SE/; accessed on 6 October 2021), but tRNA sequences could be identified neither in vB_YenMP281 nor in vB_YenP-Rambo. Phylogenetic analyses were performed using the CSI Phylogeny tool (version 1.4; https://cge.cbs.dtu.dk/services/CSIPhylogeny/; accessed on 6 October 2021) of the Center for Genomic Epidemiology [49]. If not otherwise indicated, default settings were used for the analyses. Dot plot illustrations were conducted using DS Gene (version 2.5; Accelrys Inc., San Diego, CA, USA) with parameters specified in the legends of the illustrations.

### 4.6. Nucleotide Sequencing Data

The nucleotide sequences of the phages were deposited in GenBank under the accession numbers OK042080 (vB_YenP_Rambo) and MT366944 (vB_YenM_P281).

## 5. Conclusions

Phages can be a promising tool to reduce important pathogens like *Y. enterocolitica* along the food chain. Virulent *Y. enterocolitica* phages have yet been mostly isolated from pigs or pork. This study showed that game animals may also be a valuable source of new *Yersinia* phages. Besides a large number of other phages, we isolated the podovirus vB_YenP_Rambo and the PY100-like myovirus vB_YenM_P281, both of which exhibited a wide host range and strong lytic activity. The study also suggests that *Yersinia* species are widespread in game animals, who therefore may form a reservoir for these bacteria. This assumption is corroborated by the fact that some phages were isolated from tonsils that are known to be highly populated by *Y. enterocolitica*, particularly in pigs. We will therefore continue our work to isolate phages from game animals, which can be used for various applications or might have other interesting properties.

## Figures and Tables

**Table 1:** Host range of vB_YenM_P281 and vB_YenP_Rambo on *Y. enterocolitica* strains using lysates containing approximately 10^7^ pfu/mL.

*Y. enterocolitica*	vB_YenM_P281 28 °C	vB_YenM_P281 37 °C	vB_YenP_Rambo 28 °C	vB_YenP_Rambo 37 °C
B4/O:3 (*n* = 18)	18	16	16	18
B2/O:9 (*n* = 19)	0	18	19	19
B2/O:5,27 (*n* = 17)	15	17	15	17
1B/O:8 (*n* = 8)	7	8	0	0
**Total (*n* = 62)**	**40 (62)**	**59 (62)**	**50 (62)**	**54 (62)**
***Y. enterocolitica*** **(biovar 1A)**	**vB_YenM_P281** **28 °C**	**vB_YenM_P281** **37 °C**	**vB_YenP_Rambo** **28 °C**	**vB_YenP_Rambo** **37 °C**
O:5 (YE17/18)	−	+++	−	+++
O:5 (YE65/14)	++	+++	+	+++
O:5 (YE129/15)	−	+	−	−
O:6,31 (YE30/12)	++	+++	−	+++
O:7,8 (YE21/12)	−	+++	−	−
O:7,8 (YE29/16)	−	++	−	−
O:8 (YE03/13)	−	++	−	−
O:13,17 (YE15/07)	−	+++	−	++
O:27 (YE80/16)	+++	+++	+	+++
O:27 (YE83/16)	+	++	−	+++
**Total (*n* = 10)**	**4 (10)**	**10 (10)**	**2 (10)**	**6 (10)**

Note: +++—strong lytic activity (single plaques obtained with 10^−4^ and 10^−5^ dilution), ++—medium lytic activity (single plaques obtained with 10^−2^ and 10^−3^ dilution), +—weak lytic activity (single plaques obtained with 10^−1^ dilution or an undiluted lysate), −—no lytic activity.

**Table 2:** Lytic activity of vB_YenM_P281 and PY100 on *Y. pseudotuberculosis* in the presence of calcium and magnesium cations.

*Y. pseudotuberculosis*	vB_YenM_P281 28 °C	vB_YenM_P281 37 °C	PY100 28 °C	PY100 37 °C
O:1a (*n* = 12)	7	10	8	10
O:1b (*n* = 8)	3	8	7	8
O:2a (*n* = 2)	0	0	0	0
O:2b (*n* = 1)	1	1	1	1
O:3 (*n* = 2)	1	1	2	2
O:4b (*n* = 1)	1	1	1	1
O:5a (*n* = 2)	0	1	0	1
O:6 (*n* = 4)	1	3	3	3
O:9 (*n* = 2)	1	2	1	1
Unknown O-type (*n* = 21)	16	19	18	20
**Total (*n* = 55)**	**31 (55)**	**46 (55)**	**41 (55)**	**47 (55)**

**Table 3:** Lysis of other *Yersinia* species by vB_YenP_Rambo and vB_YenM_P281 at 37 °C using lysates containing approximately 10^7^ pfu/mL.

*Yersinia* Species	Strain ID	VB_YenP_Rambo	VB_YenM_P281
*Y. intermedia*	DSM 18517	−	−
*Y. frederiksenii*	DSM 18490	+++	+
*Y. bercovieri*	DSM 18528	+++	+
*Y. similis*	DSM 18211	−	−
*Y. wautersii*	DSM 27350	+ (S)	+++ (S)
*Y. aldovae*	DSM 18303	−	−
*Y. aleksiciae*	DSM 14987	−	−
*Y. pekkanenii*	DSM 22770	−	−
*Y. rohdei*	DSM 18270	−	−
*Y. masssiliensis*	DSM 21859	−	−
*Y. kristensenii*	DSM 18543	+++	+ (S)
*Y. nurmii*	DSM 22297	−	−
*Y. ruckeri*	DSM 18506	−	−
*Y. mollaretii*	DSM 18520	−	−
**Total (** ***n*** ** = 14)**		**4 (14)**	**4 (14)**

Note: +++—strong lytic activity (single plaques obtained with 10^−4^ and 10^−5^ dilution), +—weak lytic activity (single plaques obtained with 10^−1^ dilution or an undiluted lysate), −—no lytic activity, (S)—plaques only obtained in the presence of calcium and magnesium cations (see text for details).

## Data Availability

The nucleotide sequences of the phages are available in Genbank under accession numbers as indicated above.

## Supplementary Materials

The following are available online at https://www.mdpi.com/article/10.3390/ijms222111381/s1.
