# Supplementary figures and images for: Properties of Two Broad Host Range Phages of Yersinia enterocolitica Isolated from Wild Animals
Source: Int J Mol Sci. 2021 Oct 21;22(21):11381. doi: 10.3390/ijms222111381 (PMC8583763; doi:10.3390/ijms222111381)

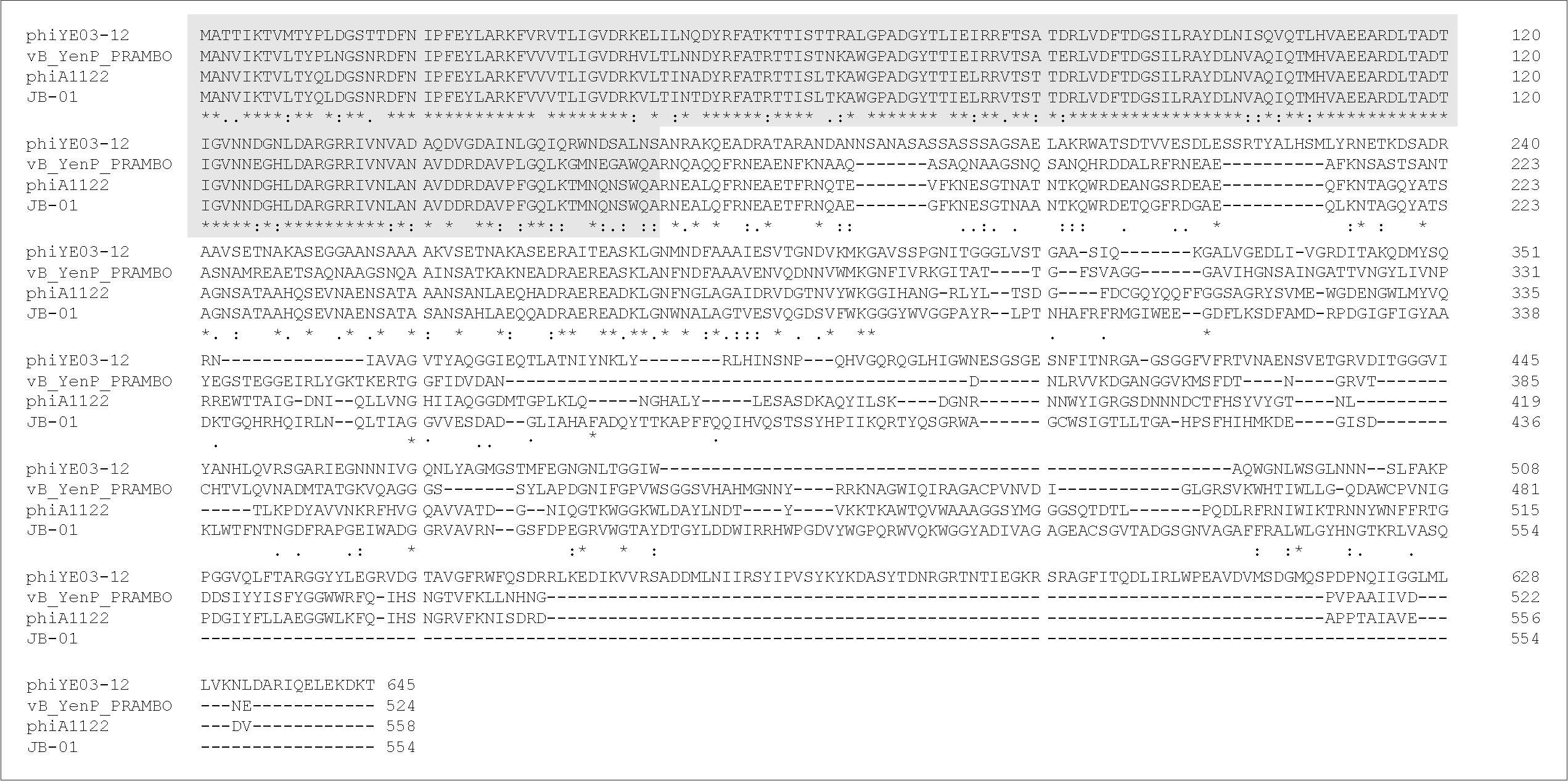

Supplement: Supplementary file 1 [file ijms-22-11381-s001.zip › FIGURE-S01.tif]

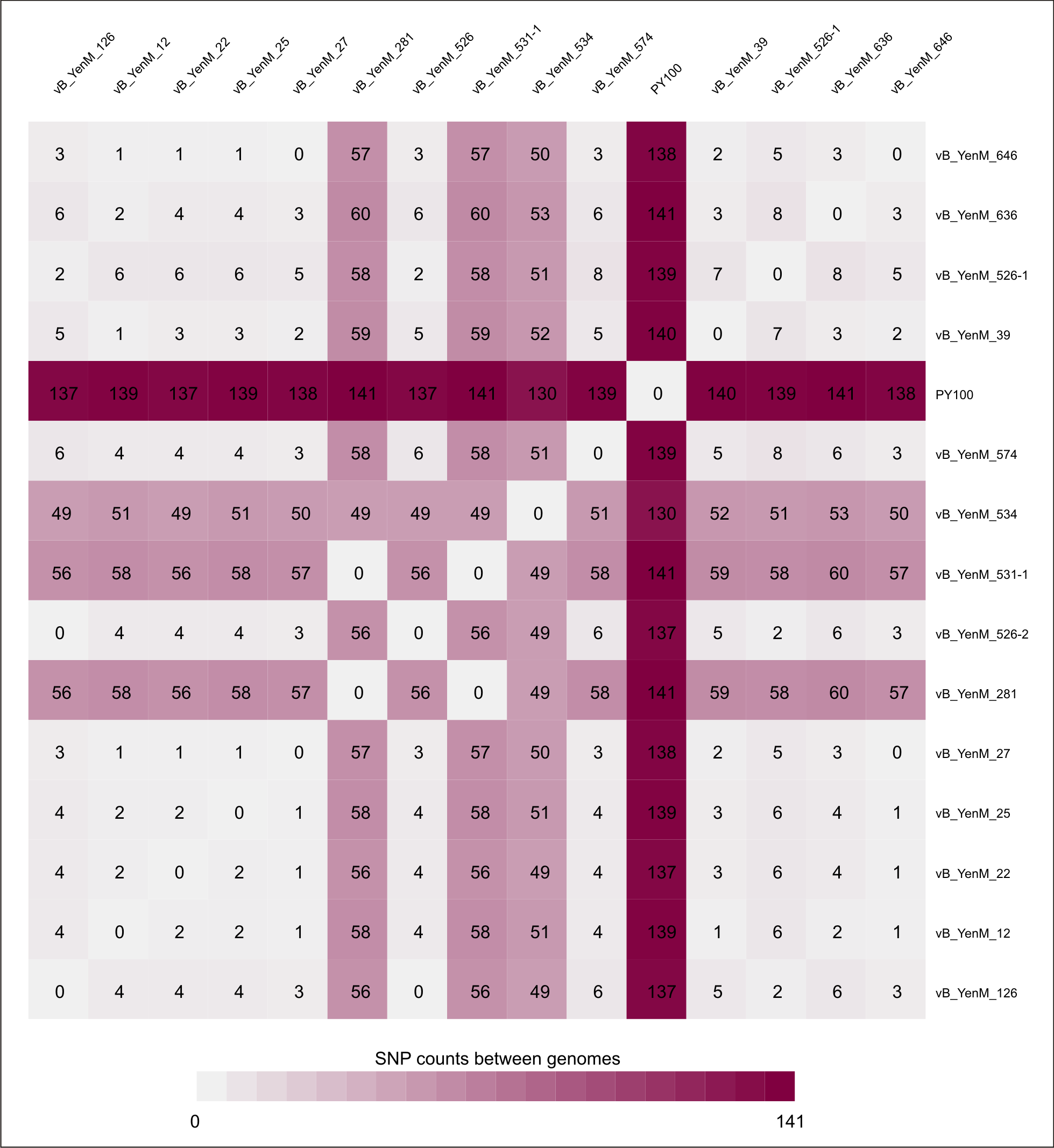

Supplement: Supplementary file 1 [file ijms-22-11381-s001.zip › FIGURE-S02.tif]
